# A knowledge-based equation of daily work exposure

**DOI:** 10.1371/journal.pone.0324924

**Published:** 2025-06-10

**Authors:** Danuta Roman-Liu, Joanna Kamińska, Tomasz Tokarski

**Affiliations:** 1 Central Institute for Labour Protection – National Research Institute (CIOP-PIB), Warsaw, Poland; Prince Sattam bin Abdulaziz University, SAUDI ARABIA

## Abstract

The objective of this study was to establish a predictive equation that expresses the daily work exposure as a function of variables that define complex work tasks. The equation was verified with data reported in reviewed publications. The ScienceDirect, PubMed, and ProQuest databases were searched using keywords related to variables that characterize intermittent tasks and those that describe muscle fatigue resulting from these tasks. Inclusion and exclusion criteria were established to focus on task characteristics and study participants. The primary criterion for accepting studies was a quantitative definition of the tasks performed, specifically concerning the level of force exerted over a specified period. Only studies involving healthy individuals aged 18–70 years that reported voluntary muscle contractions were deemed eligible. The adjustment of the prediction equation was based on the assumptions that for the same values of variables that characterize work, the exposure calculated by the equation is equivalent to musculoskeletal load, and that the level of musculoskeletal load at a given time determines the experimentally measured decrease in force capabilities. Thirty-seven datasets of independent variables (those that define work tasks) and dependent variable relevant to the decrease in force capabilities were accepted to establish the equation. Based on the comparison of dependent data from experimental studies with data obtained from calculations using independent variables, the equation that provided the best fit was identified. The correlation between the calculations and experimental results was found to be 0.72. The equation distinguishes work tasks by considering variables such as relative force, time of task, mean exposure, and the similarity of tasks performed throughout the daily work. It provides a tool for determining the work exposure associated with a specific set of tasks, which may cover the entire work shift or only parts of it.

## Introduction

Physical work is common in everyday life and at industrial sites. Workers perform tasks ranging from simple grasps to complex dexterous activities daily. Some of them are physically demanding with overuse affecting performance efficiency [1]. Overly strenuous activities impact on musculoskeletal load and may cause cumulative trauma in the structure of the body. Overload of the musculoskeletal system is considered a risk factor for injury and, to the extent, of the development of musculoskeletal disorders [2,3]. In this context, exposure associated with work plays an important role [4].

It is well acknowledged that exposure is related to the tasks performed at work and is determined by body posture, exerted force and work pace [5,6,7]. Muscle load, associated with exposure of work, plays an important role in development of work-related localised muscle fatigue [8]. The load on the musculoskeletal system that the employee experiences as a result of exposure also depends on his strength and performance capabilities [9].

Local muscle fatigue is underpinned by multiple complex mechanisms [10] and it is a known precursor to a number of negative short- and long- term implications. In the short-term perspective, fatigue is associated with decrease in strength capabilities [11]. Loss of strength depends on both the exposure associated with the task performed and with its duration, and can be described as a time-dependent reduction in the force capacity of a muscle or muscle group [12]. As a long-term outcome, localised fatigue has been linked to the development of musculoskeletal disorders [13,14]. Examples of work-related musculoskeletal disorders may include carpal tunnel syndrome [5], epicondylitis [15], rotator cuff disorders [16] and low back pain [17].

Muscle load and fatigue can be assessed through the application of experimental methods that detect the physiological response to exposure [18,19]. Local muscle fatigue can be evaluated by tracking changes in the parameters characterizing a muscle group’s electromyographic signals across time while work-related effort is performed [20]. Another technique to gauge muscular fatigue is to measure the amount of force that has been lost from the muscles group while doing specific tasks [12] or the endurance time, which represents the maximum time during which a particular effort can be maintained [21]. Assessments through the application of experimental methods are linked to particular workplace, which means that there is not one generic assessment that can be applied.

Modelling fatigue or exposure as dependent on variables that characterize work in respect to working postures, exerted forces and repetitions seems more efficient and may have more general and broader application. Therefore, research is looking for a relationship between exposure variables and fatigue. The most common and frequently cited model is that related to maximum endurance time as a measure of fatigue.

Prolonged exertions at the same force level have been studied for the development of fatigue. The study of the duration a subject could maintain a given force level [22] demonstrated the relationship between the percentage of maximum voluntary contraction (MVC) and time. Following those studies, others have determined endurance time as a function of the levels of MVC for different muscle groups [23,24]. As a consequence the relationship between the endurance time and isometric level of muscle effort is quite well established, mostly as an exponential function of the voluntary maximum contraction or the relative force required by the task [25]. There are significant differences among the models expressing the maximum endurance time for isometric exertions developed by different researchers as result of experimental studies. A detailed review of those models was performed by El ahrache et al. [26]. Differences between those models may primarily be attributed to the experimental conditions (methods used, inclusion of endurance limit, individual differences in study participants) and to the way the model is constructed [25,26]. It should be noted, however, that, long-term static loading occurs relatively rarely in real work situations, which limits the applicability of models expressing endurance time as a function of long-term isometric strength level [27].

Much less is known about how intermittent effort at submaximal levels influences the development of muscle fatigue. Intermittent strength efforts are considered to be less fatiguing compared to sustained efforts at the same level [28,29]. However, there might be some doubts if lower fatigue is a consequence of intermittent character of load or muscle fatigue development is affected by the overall effort and not the intermittent characteristics [30,31]. One may assume that if different muscle exertions have the same overall workload, the development of muscle fatigue should be the same. However, Seghers and Spaepen [32] stated that it was unclear what effect different loading patterns with the same overall mean load would have. To maintain the same mean muscle load across conditions would require manipulating variables that describe performed tasks in a systematic manner and for that purpose solid models are required. It leads to the conclusion that model which would determine exposure as a function of variables characterising complex work is needed [33,34]. Establishing such a relationship would allow for the optimization of work related load and fatigue through the optimization of exposure.

Models of relationship between the endurance time and constant load on a determined level or even intermittent load at two or three levels provide important data. However, those models are hardly applicable for assessment of work related exposure in real work places, especially when job rotation becomes one of the main protective measures against the development of musculoskeletal disorders [35]. In complex production systems, manual operations still dominate, hence work tasks are affected by high variability determined by various levels of force and various postures. As such, reliable models of exposure are dependent on the specific characteristics of any workplace, and are dearly needed. Roman-Liu [36] proposed an equation that calculates exposure related to the performance of a repetitive task using the upper limbs, which is defined by cycle duration, mean load and the number of cycle phases. Taking into account that the equation only refers to repetitive efforts and does not consider exposure of daily work or include the duration of breaks, it would be necessary to modify the equation in such a way that it becomes applicable to general working conditions. In this context, the aim of this study was to enlarge the concept proposed by Roman-Liu [36] by presenting a model for daily work exposure. Hence, a predictive equation was proposed and verified that expresses an exposure index associated with daily work as a function of variables that characterize each task performed as part of a job.

The paper’s content can be separated into a theoretical and an experimental sections. The theoretical section outlines the concept of work tasks, the variables that define work tasks and an equation that calculates the exposure index as a function of these variables. The experimental section presents the findings of a comparative analysis between model calculations and already published experimental data. Additionally included is an analysis of the variations in the exposure depending on how tasks are structured throughout the working day.

## Methods

### Exposure variables

**Fig 1:**
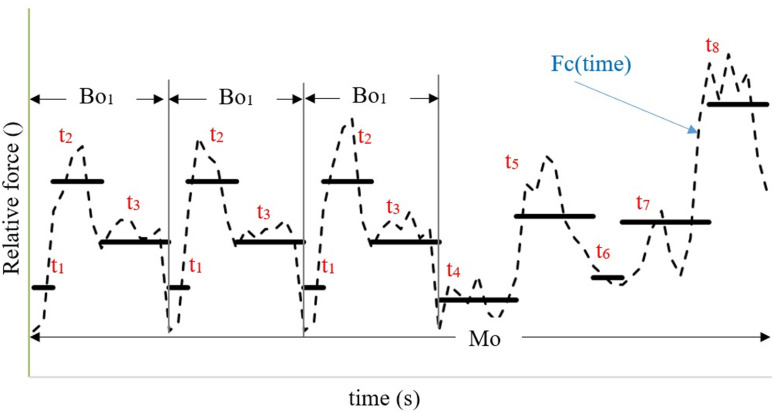
Changes in relative force as a function of time. Relative force is expressed as a continuous quantity Fc(time) and as quasi-static representation of force with a different constant values for successive task (t). Sequence of tasks create basic operations (Bo), and the main operation (Mo).

Exposure was proven to stem mostly from physical factors, namely posture and force applied during any physical activity at a given point in time. Changes in the variables of either of these two factors also change the magnitude of exposure. Typically, posture is determined by the angles at the joints, and exerted force is determined by its type and absolute value. The force can also be expressed as a relative value, calculated as the ratio of the absolute exerted force to the MVC for the same type of force and body posture. Thus, the variables that determine posture, the amount and the type of force exerted, are expressed by one variable: relative force. Commonly, however, a daily work consists of numerous changes in body posture and/or exerted force. This implies changes in muscles load, and can be described as a continuous function of time or as a quasi-static function. In the latter case, a continuous function is sampled and the duration and corresponding value of the load are assigned to individual samples, namely tasks (Fig 1).

Daily work consists of main operations (Mo) that include basic operations (Bo) and each basic operation consists of a sequence of tasks. A daily work may consist of only one basic operation repeated over work shift. In this case, the Mo is the same as the Bo ones. Most often, however, a daily work consists of many Bo. Some of the Bo may be repeated when they occur one after another or they may occur many times in different sequences. Similarly, Mo which make up daily work may be repeated. It may also be the case that daily work consists only of one operation, which is a unique sequence of tasks that cover the entire work shift. Tasks, Bo and Mo are described by several variables.

Each task is assigned the level of relative force of the task (RF) and a duration of this task. These two variables are treated as primary exposure variables and are consistent with those presented for repeated efforts in the study by Roman-Liu [36] and earlier studies by Mathiassen and Winkel [37]. On the basis of those two primary variables it is possible to calculate a few more (secondary variables), including cumulative time of work duration, which is equivalent to the total work time and is calculated as the sum of the task duration. Work duration corresponds to the cycle time outlined in the aforementioned studies [36,37]. The cyclic work discussed in the research by Mathiassen and Winkel [36] was built on a cycle made up of two tasks. The study by Roman-Liu [36] describes a model as being defined by the quantity of tasks in a single cycle. In presented hear model work is characterized by the total number of tasks thought the working time that is under analysis. Mean exposure (ME) is calculated as the sum of task RF multiplied by its duration, and then divided by the sum of tasks duration (Eq 1). The variable ME is equivalent to the average load [37] or integrated cycle load [36]. Variables in addition to those discussed previously in the aforementioned studies are relative time (RT) and task similarity (SP). RT illustrates the mean duration of one task and is calculated with Eq 2. For assessment of exposure, it is also important to note how similar or different are the successive tasks in terms of RF. The SP calculations are based on the sum of two numbers related to the number of tasks with the same RF (Eq 3). The first number expresses the number of times that the most frequent task occurs. The latter number expresses the second most common number of the same tasks. Table 1 presents summary of the exposure variables and the relationships between them.

**Table 1:** Summary of exposure variables that characterize tasks and operations and that are crucial for the assessment of daily work exposure.

Primary exposure variables (input data)	
Task (t)	RF (relative force); TT (time of duration); s_1_ (the number of times that the most frequent task occurs); s_2_ (the second most common number of the same tasks)
Basic operation (Bo)	grouping t into sequence; Nb – number of times the basic operation is repeated
Main operation (Mo)	grouping Bo into sequence; Nm – number of times the main operation is repeated
**Secondary exposure variables (calculated)**	
k – the number of all t in the daily work, taking into account Nb and Nm	
CT – cumulative time of work duration (the sum of TT of all t taking into account number of repetitions of each operation)	
ME – mean exposure (the sum of RF multiplied by TT of all t, divided by CT)	
RT – relative time = CT/k	
SP – tasks similarity = 1 + 0.5·(s_1_ + s_2_)/k	


$$\begin{array}{c} \text{ME}=\;\frac{\sum_{\text{i}=1}^{\text{k}}{{\text{RF}}_{\text{i}}\text{TT}}_{\text{i}}}{\sum_{\text{i}=1}^{\text{k}}{\text{TT}}_{\text{i}}}\;\;\;\;\;\;\;\;\;\;\;\;\;\;\;\;\;\;\;\;\;\;\;\;\;\;\;\;\;\;\;\;\;\;\;\;\; \end{array}$$ (1)

where:

ME – mean exposure

RF_i_ – relative force of an i-th task

TT_i_ – duration of an i-th task


$$\begin{array}{c} \text{RT}=\;\frac{\sum_{\text{i}=1}^{\text{k}}{\text{TT}}_{\text{i}}}{\text{k}}\;\;\;\;\;\;\;\;\;\;\;\;\;\;\;\;\;\;\;\;\;\;\;\;\;\;\;\;\;\;\;\;\;\;\;\;\;\;\;\; \end{array}$$ (2)

where:

RT – relative time

TT_i_ – duration of an i-th task

k – number of tasks


$$\begin{array}{c} \text{SP}=\;\frac{1+0.5*({\text{s}}_{1}+{\text{s}}_{2})}{\text{k}}\;\;\;\;\;\;\;\;\;\;\;\;\;\;\;\;\;\;\;\;\;\;\;\;\;\;\;\;\; \end{array}$$ (3)

where:

SP – task similarity

s_1_ – the first largest number of the same task

s_2_ – the second largest number of the same task

k – number of tasks

In summary, the presented model expands on the task variables of the previous model dedicated to repetitive tasks of upper limbs [36] by adding the concepts of: relative time of task, task similarity and operation. Exposure can be calculated in relation to one operation, a set of operations or daily work. The modified equation was verified by results from experimental studies that present force capability decrease as a reaction to exposure.

### Search strategy of relevant publications for equation modification

The search was performed in selected electronic databases, namely: ScienceDirect, PubMed and ProQuest. Keywords, abstracts and titles were scanned for the following, individually and in combinations: resumption time, endurance time, work-rest cycle, load, force, strength, torque, intermittent, fatigue, voluntary, activation, contraction, decline, decrease, drop, exhaustion, sustain. Additionally, reference lists of analysed papers, including review ones, were scanned to identify non-indexed papers that might meet the eligibility criteria.

Relevant studies were identified, synthesized and analysed culminating in a comprehensive description of changes in exerted force with time, which was defined as local fatigue associated with performed tasks. The predictive equation being the aim of analysis, done on the reviewed studies, is dedicated to the assessment of exposure that is related to the performance of work of specific characteristics. Therefore, inclusion and exclusion criteria were set to focus on task characteristics and working-age of study participants. Thus, the main criterion for accepting studies was a quantitative definition of the task performed in terms of the level of force exerted over a given period of time. Only studies that reported voluntary muscle contractions and were carried out on individuals without impairments that could affect the performance were included. This implies that the eligible studies showed variations in force capabilities over time during task execution, along with a clear description of the tasks in terms of their RF levels and corresponding durations. Only studies involving healthy individuals aged 18–70 years were considered eligible. If different variants were presented in a paper and at least one of the variants was eligible, the study was qualified for analysis. Excluded were studies that comprised any type of electrical muscle stimulation introduced during performance or were done on patients with neurodegenerative illnesses. An important criterion for excluding a paper from the analysis was the exertion of dynamic force.

### Data analysis verifying the modified equation

The main aim behind the analysis of experimental data was to determine the mathematical equation of exposure, expressed quantitatively by the Work Exposure Index (WEI), as a function of exposure variables. In order to achieve this goal, an equation developed in earlier studies [36] was modified, interrogating the best fit between the WEI equation and experimental data. Data from the literature had to be combined in order to verify the equation. Analysis was based on the assumption that exposure related to a performed task calculated by WEI, as a function of exposure variables, is equivalent to the load on the musculoskeletal system of the experimental participants. The level of musculoskeletal load (ML) at a given time determines the decrease in force capabilities measured experimentally. Therefore, ML was treated as a dependent variable derived from empirical studies.

The nature of the published data used for the equation verification precludes the standard way of combining means and standard deviations from different studies. Usually, the consolidation of data deals with a situation where pooled variables are of the same type (e.g., endurance time). In cases where a value of a variable changes over time, there are pooled those values that were recorded at the same moment in time. In analysis presented in this paper the reviewed studies differ not only in populations and test methods, but also in the duration of the experiment and time span of force measurements differed among studies. There were experiments with a given effort completion time and tests with endurance time. Both endurance time and effort completion time have been defined differently in different studies. Common among studies was that they reported set of values of force assigned to time over different time schedules. That allowed for approximation of changes in force capabilities over time via a logarithmic function. This function is specific for each case reported in each study and after normalization allows the comparison of data.

The first step in the analysis was to determine, for each study case, the regression logarithmic function expressing decrease in force capacity (FC) during the exposure (the performance of tasks) as a function of time (Eq 4).


FC=A−−B*ln(time) (4)

where:

FC – force capacity during the performance of tasks

A and B – coefficients of logarithmic function specific for each variant of study

time – duration of performance of task on load B

**Fig 2:**
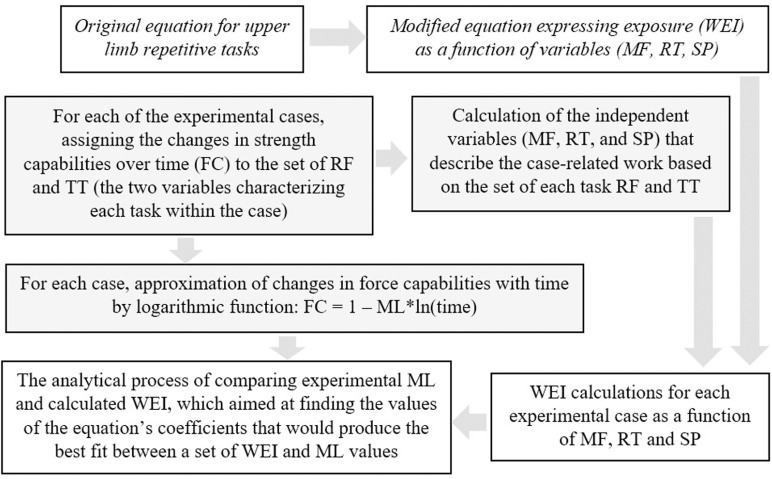
Diagram presenting the steps of process of modification and verification of the predictive equation of Work Exposure Index (WEI). FC – decrease in force capabilities, ML – musculoskeletal load, ME – mean exposure, RT – relative time of work, RF – relative force of single task, TT – time of single task, SP – task similarity.

The A and B coefficients differed between study cases. In order to normalize, the logarithmic function was transformed in such a way so that the constant value of the function, expressing the first measurement (coefficient A), equals to 100 and the B coefficient expressing decrease in force capabilities that equals to ML. Studies presented variables that characterized performed task in a way that allowed for its description in terms of the exposure variables (Table 1). Based on set of those two variables (primary exposure variables), for each study case secondary exposure variables (ME, RT and SP) were calculated with equations from 1 to 3. As a result was obtained sets of values of coefficients standing in front of the logarithm (ML) assigned to a set of ME, RT and SP values. Calculations of WEI, that was compared to ML, were performed as a function of variables ME, RT, SP specified for each case identified from experimental studies (Fig 2).

Statistical analysis was performed with the package Statistica 10. The analytical regression analysis focused on finding the best fit between ML and WEI with ME, RT, SP datasets. The Spearman correlation coefficients were calculated for comparison in order to test the relationship between the calculations performed with the model and experimental data (namely WEI and ML). The best fit determined using the linear regression function and Spearman’s R yielded the WEI equation.

## Results

### Characteristics of the studies and data extraction

**Fig 3:**
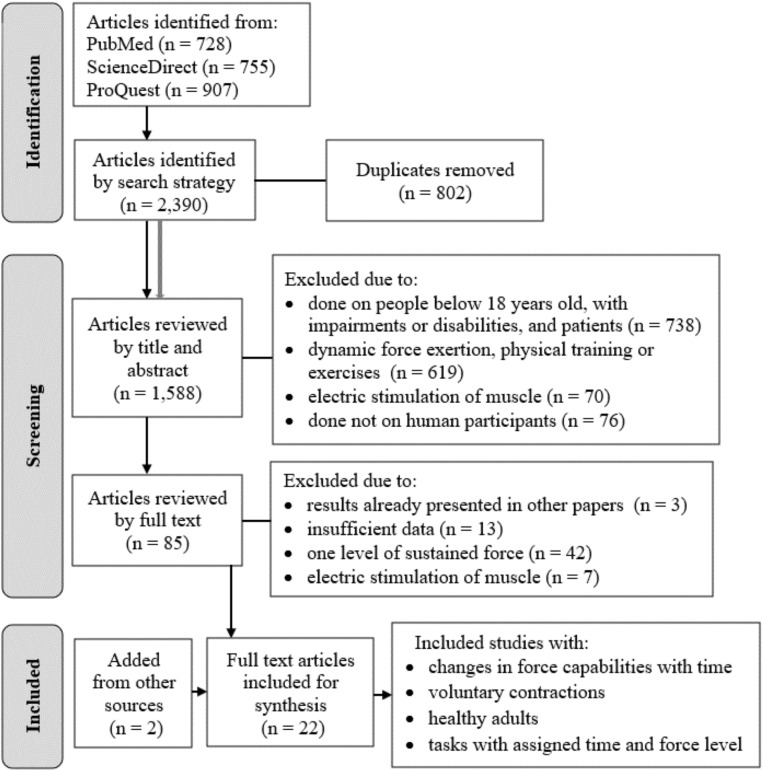
Diagram presenting the search process.

The systematic search identified 2,390 potentially eligible studies (Fig 3). Of these, 728 were obtained from PubMed, 755 from ScienceDirect, and 907 from ProQuest. After excluding 802 duplicate papers, 1,588 papers were screened for eligibility based on their titles and abstracts according to the specified criteria. Studies were excluded from further analysis if they involved non-human subjects (n = 70), participants under 18 years of age, or individuals with diseases, impairments, or disabilities (n = 738). Research that focused on non-isometric strength efforts, such as training or exercise (n = 619), as well as studies where strength was derived from muscle stimulation (n = 70), were also excluded. Following a full-text review, 65 additional studies were excluded due to insufficient data (n = 13), the use of only one level of continuous force (n = 42), results previously reported in other publications (n = 3), or force values derived from muscle stimulation (n = 7). Two studies were included from the reference lists of the reviewed papers. Ultimately, a total of 22 studies were included for data extraction. Based on the information selected for analysis and the aim of this review, the number of study participants and primary exposure variables were extracted from each retrieved paper.

Table 2 presents the characteristics of the study qualified for further analysis grouped according to values of variables that characterize the performed task. Data presented in the published studies included here, namely force measured in time, were organized descriptively in tables or figures. In each case, force was exerted in a given pattern for a given time with a given rest time. An in-depth detailed analysis of the research methods outlined in each of the reviewed paper formed the foundation for the work design created from tasks. The extracted data were assigned to the primary exposure variables. The work design with values assigned to the primary variables formed foundations for calculating the secondary exposure variables. The tasks were integrated in operations according to study description. Description also detailed the number of repetitions for each operation. The work design presented in Table 2 is in form of an equation, with each Bo depicted as the sum of the tasks, while the Mo is expressed as the sum of the Bo multiplied by the number of their repetitions, and optionally with additional tasks. This indicates that, for example, the equation Mo_01_ = 11*Bo + t_3_ + t_2_ should be interpreted as the Mo labelled as 01 made up of Bo (consisting of t_1_ and t_2_) repeated eleven times, with the extra tasks t3 and t2 also factored into this Mo.

**Table 2:** Independent variables that characterise the performed task derived from a study qualified for further analysis.

Study description		Tasks description			Work description						
		Primary exposure variables			Work design	Secondary exposure variables					
Reference	n	task	RF ()	TT (s)	main operation design	CT (s)	k ()	ME ()	RT ()	s_1_ ()	s_2_ ()
[38]	12	t_1_ t_2_ t_3_	0.6 0 1	3 2 3	Bo = t_1_ + t_2_; Mo_01_ = 11*Bo + t_3_ + t_2_	60	24	0.38	2.5	12	11
[39]	18	t_1_ t_2_ t_3_	0.3 0 1	3 2 3	Bo = t_1_ + t_2_; Mo_02_ = 12*Bo + t_3_ + t_2_	65	26	0.18	2.5	13	12
		t_1_ t_2_ t_3_	0.5 0 1	3 2 3	Bo = t_1_ + t_2_; Mo_03_ = 12*Bo + t_3_ + t_2_	65	26	0.29	2.5	13	12
[40]	13	t_1_ t_2_	1 0	5 5	Mo_04_ = Bo = t_1_ + t_2_	10	2	0.50	5	1	1
[41]	12	t_1_ t_2_ t_3_	0.3 0 1	3 2 3	Bo = t_1_ + t_2_ Mo_05_ = 23*Bo + t_3_ + t_2_	120	48	0.18	2.5	24	23
[42]	22	t_1_ t_2_	1 0	1.6	Mo_06_ = Bo = t_1_ + t_2_	3.2	2	0.5	1.6	1	1
[43]	14	t_1_ t_2_	1 0	3 5	Mo_07_ = Bo = t_1_ + t_2_	8	2	0.37	4	1	1
[44]	32	t_1_ t_2_	1 0	5 5	Mo_04_ = Bo = t_1_ + t_2_	10	2	0.5	5	1	1
[45]	24	t_1_ t_2_	1 0	5 5	Mo_04_ = Bo = t_1_ + t_2_	10	2	0.5	5	1	1
[46]	7	t_1_ t_2_ t_3_	0.3 0 1	2 4 2	Bo = t_1_ + t_2_ Mo_08_ = 9*Bo + t_3_ + t_2_	60	20	0.12	3	10	9
		t_1_ t_2_ t_3_	0.4 0 1	2 4 2	Bo = t_1_ + t_2_ Mo_09_ = 9*Bo + t_3_ + t_2_	60	20	0.15	3	10	9
		t_1_ t_2_ t_3_	0.5 0 1	2 4 2	Bo = t_1_ + t_2_ Mo_10_ = 9*Bo + t_3_ + t_2_	60	20	0.18	3	10	9
		t_1_ t_2_ t_3_	0.6 0 1	2 4 2	Bo = t_1_ + t_2_ Mo_11_ = 9*Bo + t_3_ + t_2_	60	20	0.21	3	10	9
		t_1_ t_2_ t_3_	0.8 0 1	2 4 2	Bo = t_1_ + t_2_ Mo_12_ = 4*Bo + t_3_ + t_2_	30	10	0.28	3	5	4
		t_1_ t_2_ t_3_	0.9 0 1	2 4 2	Bo = t_1_ + t_2_ Mo_13_ = 4* Bo + t_3_ + t_2_	30	10	0.30	3	5	4
[47]	8	t_1_ t_2_ t_3_	0.5 0 1	5 5 5	Bo = t_1_ + t_2_ Mo_14_ = 5*Bo + t_3_ + t_2_	60	12	0.29	5	6	5
[48]	22	t_1_ t_2_ t_3_	0.5 0 1	5 5 5	Bo = t_1_ + t_2_ Mo_15_ = 11*Bo + t_3_ + t_2_	120	24	0.27	5	12	11
[49]	8	t_1_ t_2_	1 0	5 3	Mo_16_ = Bo = t_1_ + t_2_	8	2	0.62	4	1	1
[50]	7	t_1_ t_2_	1 0	2 2	Bo = t_1_ + t_2_ Mo_17_ = 5*(10*Bo + 3*t_2_) + 10*Bo	270	120	0.22	2.25	60	60
[51]	12	t_1_ t_2_	1 0	1 1	Mo_18_ = Bo = t_1_ + t_2_	2	2	0.50	1	1	1
[52]	10	t_1_ t_2_	1 0	4 15	Mo_19_ = Bo = t_1_ + t_2_	19	2	0.21	9.5	1	1
[53]	27	t_1_ t_2_	1 0	1 1	Mo_20_ = Bo = t_1_ + t_2_	2	2	0.5	1	1	1
[12]	10	t_1_ t_2_	0.5 0	4 1	Mo_21_ = Bo = t_1_ + t_2_	5	2	0.4	2.5	1	1
		t_1_ t_2_	0.5 0.13	1 6.8	Mo_22_ = Bo = t_1_ + t_2_	7.8	2	0.18	3.9	1	1
		t_1_ t_2_	0.51 0.23	2 2	Mo_23_ = Bo = t_1_ + t_2_	4	2	0.37	2	1	1
		t_1_ t_2_	0.31 0	1 2	Mo_24_ = Bo = t_1_ + t_2_	3	2	0.1	1.5	1	1
		t_1_ t_2_	1 0	1 1	Mo_20_ = Bo = t_1_ + t_2_	2	2	0.5	1	1	1
		t_1_ t_2_	0.7 0	7 24	Mo_25_ = Bo = t_1_ + t_2_	31	2	0.16	15,5	1	1
		t_1_ t_2_	0.3 0	1 9.5	Mo_26_ = Bo = t_1_ + t_2_	10.5	2	0.03	5,25	1	1
		t_1_ t_2_	0.46 0.14	1 14.8	Mo_27_ = Bo = t_1_ + t_2_	15.8	2	0.16	7.9	1	1
		t_1_ t_2_	0.3 0.1	5 2.4	Mo_28_ = Bo = t_1_ + t_2_	7.4	2	0.24	3.7	1	1
		t_1_ t_2_	0.5 0	7 2	Mo_29_ = Bo = t_1_ + t_2_	9	2	0.39	4.5	1	1
[54]	16	t_1_ t_2_	1 0	5 5	Mo_4_ = Bo = t_1_ + t_2_	10	2	0.5	5	1	1
[55]	8	t_1_ t_2_	0.4 0	6 4	Mo_30_ = Bo = t_1_ + t_2_	10	2	0.24	5	1	1
[56]	4	t_1_ t_2_ t_3_	0.5 0 1	6 4 6	Bo = t_1_ + t_2_ Mo_31_ = 3*(t_3_ + t_2_ + 4* Bo) +t_3_	156	31	0.38	5	15	12
[57]	60	t_1_ t_2_	1 0	2.5 1	Mo_32_ = Bo = t_1_ + t_2_	3.5	2	0.71	1.75	1	1
[58]	21	t_1_ t_2_	1 0	3 5	Mo_07_ = Bo = t_1_ + t_2_	8	2	0.37	4	1	1

Note: n – number of study participants; t_i_ – i-th task in work; RF – relative force of the task; TT – time of the task; Bo – basic operation; Mo – main operation; CT – cumulative time of work duration; k – number of tasks in the work; ME – mean exposure; RT – relative time; s_1_ – the first largest number of the same task; s_2_ – the second largest number of the same task.

Studies varied due to the type of force. The largest number of studies examine knee extension [39,40,43,44,49,51,53,55,58]. The following is handgrip which is mentioned in six publications [12,41,48,54,57,58]. Three studies [38,52,53] report elbow flexion, while elbow extension is noted in one study [56]. Four studies [42,45,46,50] focused on foot flexion (both plantar and dorsi). The force of the thumb appears once [47].

All results from the selected publications were summarized for a group of 367 people. The majority of studies were carried out on people aged between 21 and 36 years. Only two studies focused on the older age group around 70 years (28 people in total).

### Exposure as a function of task variables

Twenty-three papers provided 37 sets of data (study cases). Linking cases with the same value of ME, RT and SP across all studies resulted in 31 sets associated with the ML coefficient, which were then used for regression analysis. This analysis provided the modified equation of exposure related to task performance (Eq 5).


$$\begin{array}{c} \text{WEI = 0.02*SP}*\left[23+12*\text{ME}*\frac{(\text{ME}+0.05)*(\text{RT}+30*\text{ME}{)}^{2}+280*\text{ME*RT}+200*\text{ME}}{\left(0.0002*\;{\text{RT}}^{2}+12.12*\text{ME*RT + ME}\right)}\right]\;\;\;\;\;\;\;\;\;\;\;\;\; \end{array}$$ (5)

where:

WEI – Work Exposure Index related to the performance of a given tasks

ME – mean exposure as calculated by Eq 1

RT – relative time as calculated by Eq 2 divided by 1 s

SP – tasks similarity as calculated by Eq 3

**Fig 4:**
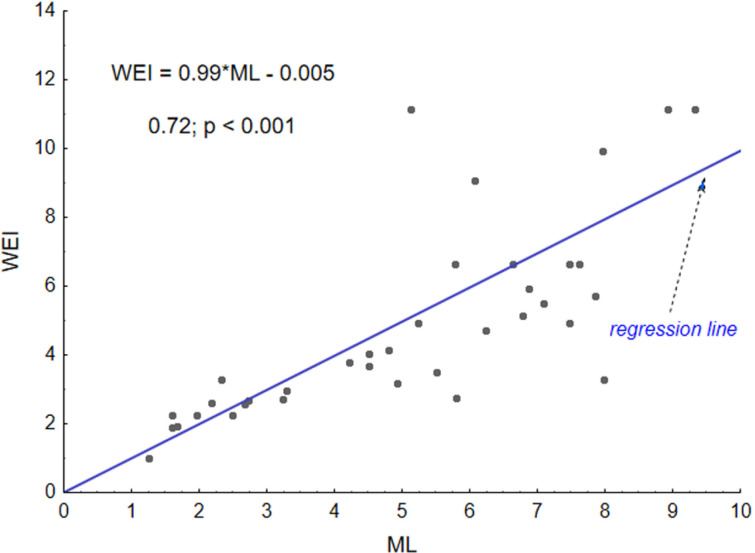
Relationship between equation calculations and experimental results. WEI – Work Exposure Index calculated with equation, ML– muscle load assessed based on results derived from reviewed studies.

All variables are dimensionless. The relationship between ML and WEI, as expressed through a linear regression function, is presented in Fig 4. The figure illustrates the relationship between independent variables from experimental studies (ML) and dependent variables (WEI), which is the result of calculations performed using the developed equation. The musculoskeletal load assessed in the experimental studies, represented by ML, arises from performing tasks defined by set of 37 exposure variables. WEI, in turn, represents the exposure calculated for each of sets of these variables. The regression line has a slope of 0.99, nearly equal to 1, and an intercept of 0.005, which is approximately zero. The Spearman correlation coefficient is 0.72 with p < 0.001. All this confirms the strong correlation between these two sets of 37 data.

The exposure is a source of fatigue that develops during task performance. Muscle fatigue can be defined as a reduction in the ability to produce force. This means that fatigue can be expressed as a decrease in force capability induced by sustained or repeated muscular contractions [8,12,59]. In this study, the decrease is quantitively expressed by Eq 4, which implies that FC can be the measure of fatigue developing under particular exposure associated with the executed work tasks. WEI assesses the level of this exposure. The analysis presented in this paper (Fig 2) proved strong correlation between ML and WEI (Fig 4). Thus, it can be inferred that WEI can act as a coefficient before the logarithmic function that describes fatigue occurring with the duration of work. This further implies that the coefficient B in Eq 4 can be replaced by WEI, assuming that the coefficient A is normalized.

**Fig 5:**
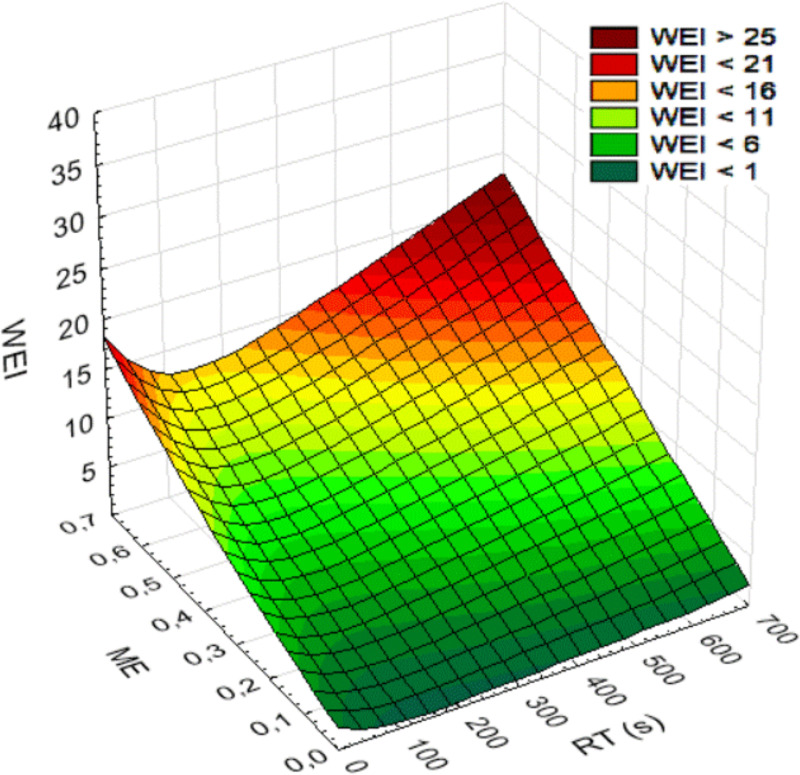
Changes in work exposure as a function of relative time and mean exposure. WEI – Work Exposure Index, RT – relative time, ME – mean exposure, task similarity (SP) equals 1.5.

**Fig 6:**
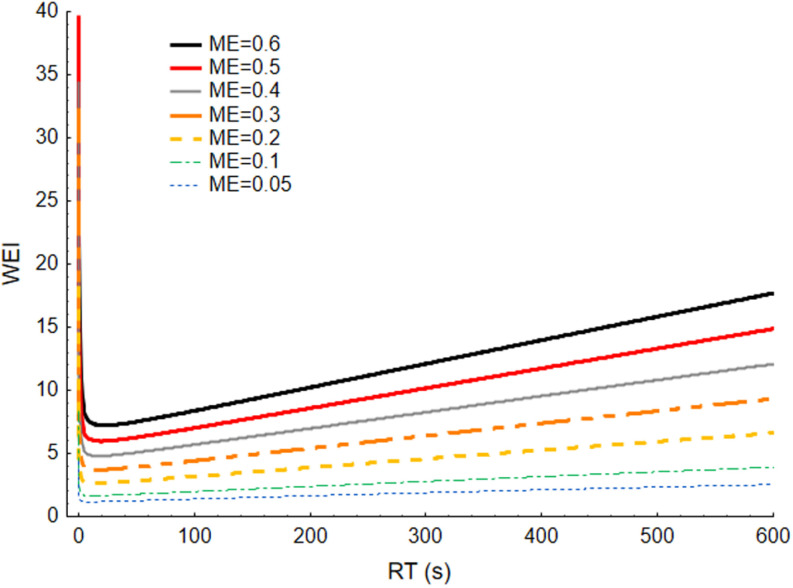
Changes in work exposure as a function of relative time for seven fixed values of mean exposure. WEI – Work Exposure Index, RT – relative time, ME – mean exposure, task similarity (SP) equals 1.5.

WEI is calculated based on two secondary exposure variables: RF and ME. To illustrate the impact of these variables on WEI, Fig 5 presents a surface graph depicting WEI as a function of RF and ME, while Fig 6 shows the changes in WEI as a function of RT for seven fixed values of ME. Both figures demonstrate the combined effect of the ME and RT variables on changes in WEI values. The lowest WEI values occur for RT ranging from a few seconds to about 50 seconds, depending on the ME. Additionally, when RF exceeds the minimum value, the increase in exposure is gradual; however, for RT values below the minimum, the increase in WEI becomes steep. This illustrates a greater increase in exposure when tasks are characterized by short durations, with a steeper increase when exertion of higher force is required. However, it is important to note that when relative force is high and task duration increases, the exposure represented by WEI also rises rapidly. An increase in relative force leads to a more pronounced rise in WEI at longer duration of tasks.

### Model utilized in real-work – example

Table 2 presents the characteristics of the Mo that were assigned to the cases in the individual studies in laboratory conditions. Many of these operations are simple two-tasks that differ only in the level of force and the duration of the individual tasks. Some of them are more complex, which means that they consist of simple Bo repeated several times and several additional tasks.

One of the main strategies used to reduce employee exposure to excessive workload is job rotation. The established mathematical equation that calculates WEI can be a good tool supporting the process of developing such a job rotation formula that would be optimal from the perspective of musculoskeletal load of all workers involved in work process. Mo characterized in Table 2 can be taken into theoretical consideration of work process optimization. Many possibilities of organizing these operations can be considered. WEI can be compared between the scenario where one employee repeats the entire set of operations, set of few selected ones and the scenario where each employee completes one main operation from Table 2 and repeats it multiple times during the workday.

**Fig 7:**
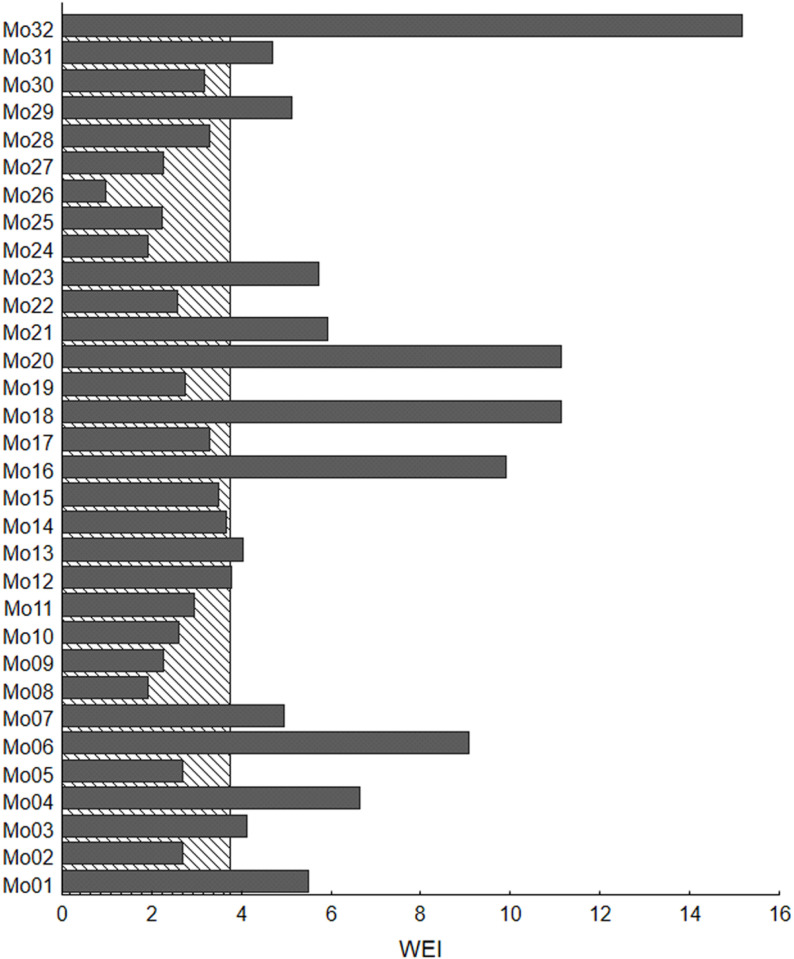
Comparison of WEI values for two scenarios. One hatched bar – every employee repeats the entire set of operations during a working day. Dark grey filled bars – every employee performs only one Mo marked as in Table 2, and repeats it multiple times during a working day.

Fig 7 presents WEI values for two scenarios. In the first scenario, only one main operation, marked in a manner analogous to that in Table 2, is performed throughout the entire working day (solid bars). In the second scenario, all operations are combined into daily work (hatched box). The WEI values for the option where one main operation is repeated throughout the workday differ significantly. The operation marked as Mo26 has the lowest WEI at 0.98, while the operation designated as Mo32 has an exceptionally high exposure of 15.17. The average WEI value for all operations is 4.57. When operations are conducted in a scenario where every worker completes a set of all operations, the WEI is 3.75, which is lower than the average WEI across all operations. In certain instances, the WEI for the combined daily work scenario is higher than that for a single operation performed during the workday. There are twelve cases where a worker would experience higher exposure in this option compared to performing only one operation, and another four cases where the WEI would be at a very similar level. However, eighteen workers would have lower exposures if each worker performs a complete set of operations. In one case (Mo32), the exposure is nearly four times lower, and in two other cases (Mo18 and Mo20), it is nearly three times lower. Additionally, two workers would have exposures that are more than twice as low (Mo06 and Mo16).

**Fig 8:**
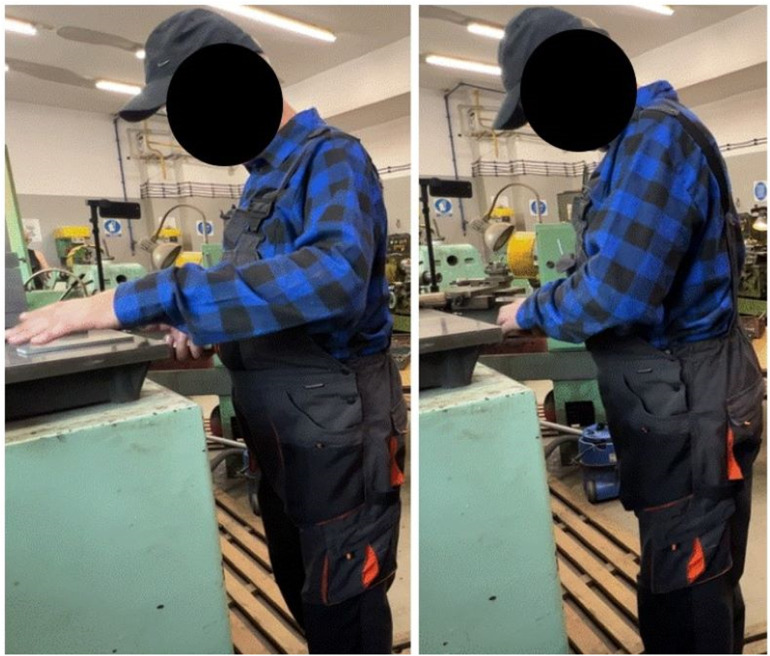
Example postures illustrating two-tasks operations of the left upper limb. Task t_grab_ is to grab the plate, task t_place_ is to put it down on the right hand.

Simple two task operations, characterized in Table 2, (Mo_04_, Mo_06_, Mo_07_, Mo_19_ – Mo_30_, Mo_32_) can be illustrated by real work of lifting plates. Based on the video recording of the work process, tasks can be identified and assigned specific body positions. The force exerted during each task, measured by its absolute or relative value, determines the RF that defines the task. Fig 8 illustrates the body positions assigned to two tasks involving the left upper limb. The first left upper limb task (t_grab_) is to grab the plate, and the second task (t_place_) is to put it down on the right hand. The force levels defined by the variable RF depend on the mass of the plate and the position of the upper limb. Both RF and task duration can correspond to those defining the above-mentioned operations.

Daily work that consist of operations repeated many times with additional tasks (Mo_01_, Mo_02_, Mo_03_, Mo_05_, Mo_08_ – Mo_15_) can be projected into real work environments by including two additional tasks to those shown in Fig 8. The Mo then consists of few Bo (tasks t_grab_ and t_place_) repeated multiple times, and additional tasks t_walkright_ and t_walkleft_, during which the employee may walk with carrying plates. The force levels, the number of repetitions of the Bo and the duration of the individual tasks may vary.

The proposed WEI is dedicated to the assessment of the load/exposure associated with the work performance at real workplaces. To calculate WEI, the values of the primary variables must first be determined. The assessment of duration of each task is relatively easy. It can be done by video recording the subsequent tasks. The RF assessment is more demanding and time-consuming. The body position and applied force constitute the unit load associated with a particular task. RF corresponds to the relative unit load, i.e., the ratio between the applied force and the MVC exerted in the same body position. Commonly, under real working conditions, applied force is known. The MVC can be obtained using two different approaches.

The first approach involves measurement of employee’s MVC that align with each work task. Measurements of MVC must be taken in the same body posture in which the task is performed. Such measurements of MVC can be challenging due to the time-consuming nature of the measurements and the disruption to workflow. The second approach focuses on the values of the body angles linked with a task. The body angle values can be estimated from a photograph illustrating the body posture. However, estimating the angles in this manner can be a time-consuming process even when employing tools such as Kinovea [60,61]. Using sensors to measure employees’ body angles during work provides quantitative data and aids in determining angles values [62,63]. Such equipment can be safely used during work without disrupting the workflow. The estimated or measured angle values serve as input data for calculations of MVC. The MVC for general population can be calculated as a function of the angles that define the position of the body with application of the previously developed mathematical relationships [64,65]. Another way to calculate the RF is to express the load in body part (for example the lumbar back) as a function of the body position angles and the applied force, and subsequently relate this load to the maximum allowable load [66].

All calculations resulting in RF can be done with support of a software package containing the mathematical equations. The software package can also calculate the secondary variables and the WEI. Such approach will allow a relatively easy assessment of the exposure level due to a series of tasks that make up an operation or a working day. That will allow comparison of exposure among different workplaces. When WEI is supported by relevant and verified criteria it can be also used for assessment of risk for development of musculoskeletal disorders.

## Discussion

The objective of this study was to establish and verify a predictive model that expresses exposure of performed task as a function of variables defining daily work and muscular fatigue as the logarithmic function of time with the exposure as a main coefficient. Muscle fatigue has been cited as a risk factor for injury in the workplace [59]. This leads to the assumption that decrease in fatigue through the application of tools that assess fatigue can be useful in a healthcare context. Models referring to fatigue or endurance time have already been established [22,67]. These were based on independent variables that characterized constant force [67] or, in very few cases, intermittent load according to a predetermined pattern [12,68]. The models were established based on fitted functions using muscular strength data from studies with a relatively small sample. The model presented in this study expresses exposure (equivalent to musculoskeletal load) as a function of ME, RT and SP, which makes it closer to real workplaces where work tasks are more complex. Additionally, the equation of exposure was verified based on data from 22 publications presenting 37 study cases, which makes it reliable for assessing load in real work environment.

Independent variables that refer to ME, relative time and the SP were chosen as most characteristic of a daily work. In spite of some degree of simplification in terms of exposure variables, they have been deemed to be the most critical variables for work exposure from one side and musculoskeletal load from the other [5]. Those variables refer to posture, force and time characteristics, taking into account differences between tasks. An early study has shown that those three factors are the main leading to the development of fatigue [69].

One of the decisions was about the number of study participants. Whether weighed or regular averages should be used was questioned. Studies differed in the number of participants, their sex and age, as well as the number of force measurements. All those features could have had an impact on the obtained results [39,44,45,49,54]. In addition, posture during measurement and the type of force may impact force capabilities [70]. Due to relative values that were under analysis, it has been decided that number of study participants will not be taken into consideration. This means that each study variant had the same weight in analysis of correlation between empirical and theoretical studies.

In the model presented here, force is expressed as a relative value. In calculating this value, both MVC and exerted force were considered for the same posture. In this way, posture could be ignored in subsequent analyses. Repetitiveness is determined by variables such as RT and SP. These characterize how often changes in RF take place and how similar tasks are, which are also crucial when work exposure is considered. The analysis was based on relative force measures presented in reviewed papers. This means that both individual factors related to sex and age, included in the measurements (type of force, engaged joint and posture), which had an impact on RF could have been ignored. As a consequence, the WEI index is not biased by individual factors and can be broadly applied.

The meta-analysis confirmed a strong correlation between the experimental studies and the calculations performed for the same variables characterizing a task. The Spearman’s correlation coefficient values were 0.72 with p < 0.001. Such a correlation is considered strong [71]. Thus, the presented model is a tool that may assist in quantifying exposure depending on the variables of task characteristics and then based on the exposure quantifying fatigue after a determined time of this exposure. Applying a model that allows for the assessment of fatigue after a predetermined period of time in industrial workplaces can lead to considerable improvements in terms of the avoidance of work-related disorders.

### Limitations of the study and future studies

Limitations of the study refer mostly to the quantity of data available for verification, which was determined by results of review. Linear regression determined the relationship between computational and empirical outcomes on the available sample size, which was 37 cases. Jenkins [72] findings recommend sample sizes for linear regression and meta-regression analyses. For data with low variance, the sample size should be greater than 8 to perform a reliable regression analysis. For data with high variance, the sample size should be greater than 25 [72]. His study does not recommend the maximum sample size. A sample size calculation for a single study using linear regression with G*Power, assuming power (1-beta) of 0.8 and alpha error of 0.05, recommends a minimum sample size of 14 [73]. Calculations using the same tool suggest that increasing the sample size to 37 samples would increase the power to 0.99. This indicates that a larger sample size reduces the margin of error and increases the statistical power to detect true effects.

Increasing the sample size in experimental studies is not always cost-effective [74]. However, when analysing data from review studies, the sample size is predetermined. Furthermore, combining the results of different studies with a certain precision and accuracy of the data can improve accuracy and reduce bias compared to a single study. The analysis presented in this paper has a larger sample size than required. Running statistical analysis on a larger combined sample might ensure that the confirmed and accepted results are more reliable and accurate. The data analysed in this paper are based on papers published in scientific journals reported in significant databases. All are original studies, the results are not cited and are not derived from each other, indicating their reliability. The extent to which a predictive model reflects reality depends on the available validation options, in other word on available results from already completed studies. However, it should be kept in mind that what is accepted as fact today may be modified based on newly reported measurements.

This review focused only on cases of voluntary contractions because it assumed that electrical stimulation could influence the pattern of fatigue, as proven for example in Behm et al. [75], who showed that unilateral transcutaneous electrical nerve stimulation prolonged time to failure. Such attitude limited the number of studies eligible for verification and, at the same time, evaded additional factors that may seriously impact study results. However, optimal verification of the equation would be achieved when large numbers of groups and sets of dependent and independent variables are included in the process of equation verification.

Due to the limited number of studies, variables related to different types of force and different joints were pooled. This is somewhat of a simplification, given that Frey Law and Avin’s [76] study of static endurance time showed that different parts of the body have different fatigue rates for the same load. Such approach allowed to verify the equation that may refer to various types of force, formulated according to reviewed publications. The studies were conducted mostly on handgrip, knee flexion, elbow flexion and extension, and ankle dorsi and planar flexion. This can be considered as an advantage of this study because usually work processes requires various types of force, which may differ in the subsequent tasks. Focusing only on one of these during model verification would be a limitation in model applications. Due to such attitude the equation can be used in cases where a sequence of tasks with various types of force are performed.

Some limitation of the study is the fact that individual factors can cause metabolic changes in muscles, resulting in a decline of muscle power output that increases with fatigue and varies in nature. The influence of many of these factors was not analysed in this study. Only variables that define the performed task were taken as meaningful. Factors that influence strength, such as muscle fibre composition (slow or fast twitch fibres), posture, individual differences, strength, gender, age, motivation and training [24,77] were not taken into account in the analysis.

The majority of the reviewed studies participants were between 21 and 36 years old. The population aged 45 years or older was only about 7% of the total population. This suggests that the proposed equation appears to be biased towards younger workforce demographics, which can be considered a certain limitation to the application of the equation. Nevertheless, studies [44,45,77] show that effort at a specific level caused a lower decline in muscle strength in the older group compared to the younger group. This confirms that people become less prone to fatigue as they get older. As a consequence of that the index calculated from the equation is likely to overestimate the load on older employees, indicating that it can be safely applied to them.

Despite the existing limitations, the presented equation can be implemented for both the assessment of any existing workplace and in the process of workplace planning and development. The presented model undertakes a wholistic approach to daily work and calculations of exposure can be performed in relation to tasks, one operation, a set of operations or daily work. Expressing work exposure as a function of ME, RT and SP provides a simple and easy-to-use model for practical application. Further study should focus on determining criteria that influence the risk of developing musculoskeletal disorders, e.g., values of WEI that define risk as high, medium or low.

## Conclusions

This study extends the knowledge on work process patterns by proposing a model of daily work exposure, defining tasks grouped into operations, and a mathematical equation that calculates exposure as a function of the variables defining each of daily work tasks. The verification of the model, based on data drawn from existing studies, showed a strong correlation between the experimental data obtained from the literature and calculations based on the model (0.72). The proposed equation can be used as a tool to determine occupational exposure during a specific set of tasks. It allows to monitor load associated to a performed work in any workplace, which would be beneficial for analyzing the risk of development of musculoskeletal disorders. The equation forms the basis for a computerized method for assessing the risk of developing musculoskeletal disorders. Assessing work-related exposure using the equation can lead to improved design of work processes.

## Supporting information

S1 Table Row data obtained from reviewed papers. The table presents the raw results of the studies described in the papers that were the focus of the reviews. These data reflect the level of force applied and the corresponding time the load was held in each case. Muscle load (ML), which was significant in the ensuing analysis, was estimated using this information.
(PDF)
