# Supplementary material for: A knowledge-based equation of daily work exposure
Source: PLoS One. 2025 Jun 10;20(6):e0324924. doi: 10.1371/journal.pone.0324924 (PMC12151403; doi:10.1371/journal.pone.0324924)
Supplement: S1 Table — The table presents the raw results of the studies described in the papers that were the focus of the reviews. These data reflect the level of force applied and the corresponding time the load was held in each case. Muscle load (ML), which was significant in the ensuing analysis, was estimated using this information. (PDF) [file pone.0324924.s001.pdf]

S1.Table. Row data obtained from reviewed papers

REFERENCES		time (%)	Force (%MVC)
Allman and Rice, 2003	Males young	1	100
Allman and Rice, 2003	Males young	24,5	81
Allman and Rice, 2003	Males young	100	56
Allman and Rice, 2003	Males old	1	100
Allman and Rice, 2003	Males old	24,5	80
Allman and Rice, 2003	Males old	100	57
		time (%)	Force (%MVC)
Ansdell et al., 2017	Males knee extension	1	100
Ansdell et al., 2017	Males knee extension	25	88
Ansdell et al., 2017	Males knee extension	50	81
Ansdell et al., 2017	Males knee extension	75	75
Ansdell et al., 2017	Males knee extension	100	68
Ansdell et al., 2017	Females knee extension	1	100
Ansdell et al., 2017	Females knee extension	25	85
Ansdell et al., 2017	Females knee extension	50	82
Ansdell et al., 2017	Females knee extension	75	82
Ansdell et al., 2017	Females knee extension	100	75
		time (%)	Torque (%MVC)
Armatas et al., 2010	Knee extension	1	100
Armatas et al., 2010	Knee extension	10	90
Armatas et al., 2010	Knee extension	20	80
Armatas et al., 2010	Knee extension	30	78
Armatas et al., 2010	Knee extension	40	76
Armatas et al., 2010	Knee extension	50	72
Armatas et al., 2010	Knee extension	60	69
Armatas et al., 2010	Knee extension	70	62
Armatas et al., 2010	Knee extension	80	56
Armatas et al., 2010	Knee extension	90	52
Armatas et al., 2010	Knee extension	100	49
		time (s)	Force (%MVC)
Benwell et al., 2007	Handgrip	1	100
Benwell et al., 2007	Handgrip	120	90
Benwell et al., 2007	Handgrip	240	86
Benwell et al., 2007	Handgrip	360	82
Benwell et al., 2007	Handgrip	480	79
Benwell et al., 2007	Handgrip	600	77

		time (s)	Force (%MVC)
Birtles et al., 2003	Foot flexion	1	100
Birtles et al., 2003	Foot flexion	60	77
Birtles et al., 2003	Foot flexion	120	71
Birtles et al., 2003	Foot flexion	180	69
Birtles et al., 2003	Foot flexion	240	69
Birtles et al., 2003	Foot flexion	300	65
Birtles et al., 2003	Foot flexion	360	65
Birtles et al., 2003	Foot flexion	420	65
Birtles et al., 2003	Foot flexion	480	66
Birtles et al., 2003	Foot flexion	540	66
Birtles et al., 2003	Foot flexion	600	66
Birtles et al., 2003	Foot flexion	660	65
Birtles et al., 2003	Foot flexion	720	66
Birtles et al., 2003	Foot flexion	780	61
Birtles et al., 2003	Foot flexion	840	62
Birtles et al., 2003	Foot flexion	900	62
Birtles et al., 2003	Foot flexion	960	60
Birtles et al., 2003	Foot flexion	1020	60
Birtles et al., 2003	Foot flexion	1080	59
Birtles et al., 2003	Foot flexion	1140	59

		time (s)	Force (%MVC)
Boccia et al., 2017	Knee extension	4	100
Boccia et al., 2017	Knee extension	16	94
Boccia et al., 2017	Knee extension	24	91
Boccia et al., 2017	Knee extension	32	90
Boccia et al., 2017	Knee extension	40	89
Boccia et al., 2017	Knee extension	48	87
Boccia et al., 2017	Knee extension	56	85
Boccia et al., 2017	Knee extension	64	84
Boccia et al., 2017	Knee extension	72	83
Boccia et al., 2017	Knee extension	80	82
Boccia et al., 2017	Knee extension	88	82
Boccia et al., 2017	Knee extension	96	80
Boccia et al., 2017	Knee extension	104	80
Boccia et al., 2017	Knee extension	112	79
Boccia et al., 2017	Knee extension	120	79

		Time (s)	Force (%MVC)
Callahan et al., 2009	Younger Knee extension	1	100
Callahan et al., 2009	Younger Knee extension	30	89
Callahan et al., 2009	Younger Knee extension	60	78
Callahan et al., 2009	Younger Knee extension	90	71
Callahan et al., 2009	Younger Knee extension	120	68
Callahan et al., 2009	Younger Knee extension	150	65

Callahan et al., 2009	Younger Knee extension	180	66
Callahan et al., 2009	Younger Knee extension	210	65
Callahan et al., 2009	Younger Knee extension	240	65
Callahan et al., 2009	Older Knee extension	1	100
Callahan et al., 2009	Older Knee extension	30	85
Callahan et al., 2009	Older Knee extension	60	74
Callahan et al., 2009	Older Knee extension	90	61
Callahan et al., 2009	Older Knee extension	120	59
Callahan et al., 2009	Older Knee extension	150	57
Callahan et al., 2009	Older Knee extension	180	72
Callahan et al., 2009	Older Knee extension	210	72
Callahan et al., 2009	Older Knee extension	240	72

		Time(s)	Force (%MVC)
Chung et al., 2007	Young Ankle dorsiflexion	1	100
Chung et al., 2007	Young Ankle dorsiflexion	10	98
Chung et al., 2007	Young Ankle dorsiflexion	20	91
Chung et al., 2007	Young Ankle dorsiflexion	30	89
Chung et al., 2007	Young Ankle dorsiflexion	40	87
Chung et al., 2007	Young Ankle dorsiflexion	50	82
Chung et al., 2007	Young Ankle dorsiflexion	60	84
Chung et al., 2007	Young Ankle dorsiflexion	70	84
Chung et al., 2007	Young Ankle dorsiflexion	80	80
Chung et al., 2007	Young Ankle dorsiflexion	90	80
Chung et al., 2007	Young Ankle dorsiflexion	100	79
Chung et al., 2007	Young Ankle dorsiflexion	110	76
Chung et al., 2007	Young Ankle dorsiflexion	120	75
Chung et al., 2007	Young Ankle dorsiflexion	130	75
Chung et al., 2007	Young Ankle dorsiflexion	140	74
Chung et al., 2007	Young Ankle dorsiflexion	150	74
Chung et al., 2007	Young Ankle dorsiflexion	160	74
Chung et al., 2007	Young Ankle dorsiflexion	170	72
Chung et al., 2007	Young Ankle dorsiflexion	180	72
Chung et al., 2007	Young Ankle dorsiflexion	190	72
Chung et al., 2007	Young Ankle dorsiflexion	200	70
Chung et al., 2007	Young Ankle dorsiflexion	210	66
Chung et al., 2007	Young Ankle dorsiflexion	220	68
Chung et al., 2007	Young Ankle dorsiflexion	230	66
Chung et al., 2007	Young Ankle dorsiflexion	240	66
Chung et al., 2007	Young Ankle dorsiflexion	250	66
Chung et al., 2007	Young Ankle dorsiflexion	260	65
Chung et al., 2007	Young Ankle dorsiflexion	270	65
Chung et al., 2007	Young Ankle dorsiflexion	280	66
Chung et al., 2007	Young Ankle dorsiflexion	290	63
Chung et al., 2007	Young Ankle dorsiflexion	300	60
Chung et al., 2007	Young Ankle dorsiflexion	310	59
Chung et al., 2007	Young Ankle dorsiflexion	320	60
Chung et al., 2007	Young Ankle dorsiflexion	330	63

Chung et al., 2007	Young Ankle dorsiflexion	340	60
Chung et al., 2007	Young Ankle dorsiflexion	350	59
Chung et al., 2007	Old Ankle dorsiflexion	1	100
Chung et al., 2007	Old Ankle dorsiflexion	10	98
Chung et al., 2007	Old Ankle dorsiflexion	20	93
Chung et al., 2007	Old Ankle dorsiflexion	30	91
Chung et al., 2007	Old Ankle dorsiflexion	40	90
Chung et al., 2007	Old Ankle dorsiflexion	50	86
Chung et al., 2007	Old Ankle dorsiflexion	60	85
Chung et al., 2007	Old Ankle dorsiflexion	70	84
Chung et al., 2007	Old Ankle dorsiflexion	80	85
Chung et al., 2007	Old Ankle dorsiflexion	90	83
Chung et al., 2007	Old Ankle dorsiflexion	100	83
Chung et al., 2007	Old Ankle dorsiflexion	110	83
Chung et al., 2007	Old Ankle dorsiflexion	120	83
Chung et al., 2007	Old Ankle dorsiflexion	130	83
Chung et al., 2007	Old Ankle dorsiflexion	140	83
Chung et al., 2007	Old Ankle dorsiflexion	150	83
Chung et al., 2007	Old Ankle dorsiflexion	160	81
Chung et al., 2007	Old Ankle dorsiflexion	170	83
Chung et al., 2007	Old Ankle dorsiflexion	180	81
Chung et al., 2007	Old Ankle dorsiflexion	190	82
Chung et al., 2007	Old Ankle dorsiflexion	200	80
Chung et al., 2007	Old Ankle dorsiflexion	210	79
Chung et al., 2007	Old Ankle dorsiflexion	220	81
Chung et al., 2007	Old Ankle dorsiflexion	230	80
Chung et al., 2007	Old Ankle dorsiflexion	240	80
Chung et al., 2007	Old Ankle dorsiflexion	250	80
Chung et al., 2007	Old Ankle dorsiflexion	260	81
Chung et al., 2007	Old Ankle dorsiflexion	270	80
Chung et al., 2007	Old Ankle dorsiflexion	280	80
Chung et al., 2007	Old Ankle dorsiflexion	290	80
Chung et al., 2007	Old Ankle dorsiflexion	300	81
Chung et al., 2007	Old Ankle dorsiflexion	310	81
Chung et al., 2007	Old Ankle dorsiflexion	320	82
Chung et al., 2007	Old Ankle dorsiflexion	330	81
Chung et al., 2007	Old Ankle dorsiflexion	340	81
Chung et al., 2007	Old Ankle dorsiflexion	350	78

		time (s)	Force (%MVC)
Egana and Green, 2007	Foot/ankle – pusł 30%MVC	1	100
Egana and Green, 2007	Foot/ankle – pusł 30%MVC	60	92
Egana and Green, 2007	Foot/ankle – pusł 30%MVC	120	92
Egana and Green, 2007	Foot/ankle – pusł 30%MVC	180	90
Egana and Green, 2007	Foot/ankle – pusł 30%MVC	240	91
Egana and Green, 2007	Foot/ankle – pusł 30%MVC	300	90
Egana and Green, 2007	Foot/ankle – pusł 30%MVC	360	91
Egana and Green, 2007	Foot/ankle – pusł 30%MVC	420	91

Egana and Green, 2007	Foot/ankle – push	30%MVC	480	90
Egana and Green, 2007	Foot/ankle – push	30%MVC	540	90
Egana and Green, 2007	Foot/ankle – push	30%MVC	600	88
Egana and Green, 2007	Foot/ankle – push	30%MVC	660	89
Egana and Green, 2007	Foot/ankle – push	30%MVC	720	88
Egana and Green, 2007	Foot/ankle – push	30%MVC	780	88
Egana and Green, 2007	Foot/ankle – push	30%MVC	840	89
Egana and Green, 2007	Foot/ankle – push	30%MVC	900	87
Egana and Green, 2007	Foot/ankle – push	30%MVC	960	89
Egana and Green, 2007	Foot/ankle – push	30%MVC	1020	89
Egana and Green, 2007	Foot/ankle – push	30%MVC	1080	87
Egana and Green, 2007	Foot/ankle – push	30%MVC	1140	88
Egana and Green, 2007	Foot/ankle – push	30%MVC	1200	87
Egana and Green, 2007	Foot/ankle – push	40%MVC	1	100
Egana and Green, 2007	Foot/ankle – push	40%MVC	60	95
Egana and Green, 2007	Foot/ankle – push	40%MVC	120	94
Egana and Green, 2007	Foot/ankle – push	40%MVC	180	92
Egana and Green, 2007	Foot/ankle – push	40%MVC	240	93
Egana and Green, 2007	Foot/ankle – push	40%MVC	300	92
Egana and Green, 2007	Foot/ankle – push	40%MVC	360	91
Egana and Green, 2007	Foot/ankle – push	40%MVC	420	90
Egana and Green, 2007	Foot/ankle – push	40%MVC	480	88
Egana and Green, 2007	Foot/ankle – push	40%MVC	540	88
Egana and Green, 2007	Foot/ankle – push	40%MVC	600	86
Egana and Green, 2007	Foot/ankle – push	40%MVC	660	87
Egana and Green, 2007	Foot/ankle – push	40%MVC	720	85
Egana and Green, 2007	Foot/ankle – push	40%MVC	780	87
Egana and Green, 2007	Foot/ankle – push	40%MVC	840	88
Egana and Green, 2007	Foot/ankle – push	40%MVC	900	88
Egana and Green, 2007	Foot/ankle – push	40%MVC	960	87
Egana and Green, 2007	Foot/ankle – push	40%MVC	1020	88
Egana and Green, 2007	Foot/ankle – push	40%MVC	1080	86
Egana and Green, 2007	Foot/ankle – push	40%MVC	1140	85
Egana and Green, 2007	Foot/ankle – push	40%MVC	1200	83
Egana and Green, 2007	Foot/ankle – push	50%MVC	1	100
Egana and Green, 2007	Foot/ankle – push	50%MVC	60	95
Egana and Green, 2007	Foot/ankle – push	50%MVC	120	94
Egana and Green, 2007	Foot/ankle – push	50%MVC	180	92
Egana and Green, 2007	Foot/ankle – push	50%MVC	240	93
Egana and Green, 2007	Foot/ankle – push	50%MVC	300	92
Egana and Green, 2007	Foot/ankle – push	50%MVC	360	91
Egana and Green, 2007	Foot/ankle – push	50%MVC	420	90
Egana and Green, 2007	Foot/ankle – push	50%MVC	480	88
Egana and Green, 2007	Foot/ankle – push	50%MVC	540	88
Egana and Green, 2007	Foot/ankle – push	50%MVC	600	86
Egana and Green, 2007	Foot/ankle – push	50%MVC	660	87
Egana and Green, 2007	Foot/ankle – push	50%MVC	720	85
Egana and Green, 2007	Foot/ankle – push	50%MVC	780	87
Egana and Green, 2007	Foot/ankle – push	50%MVC	840	88
Egana and Green, 2007	Foot/ankle – push	50%MVC	900	88

Egana and Green, 2007	Foot/ankle – push	50%MVC	960	87
Egana and Green, 2007	Foot/ankle – push	50%MVC	1020	88
Egana and Green, 2007	Foot/ankle – push	50%MVC	1080	86
Egana and Green, 2007	Foot/ankle – push	50%MVC	1140	85
Egana and Green, 2007	Foot/ankle – push	50%MVC	1200	83
Egana and Green, 2007	Foot/ankle – push	60%MVC	1	100
Egana and Green, 2007	Foot/ankle – push	60%MVC	60	93
Egana and Green, 2007	Foot/ankle – push	60%MVC	120	91
Egana and Green, 2007	Foot/ankle – push	60%MVC	180	90
Egana and Green, 2007	Foot/ankle – push	60%MVC	240	88
Egana and Green, 2007	Foot/ankle – push	60%MVC	300	88
Egana and Green, 2007	Foot/ankle – push	60%MVC	360	85
Egana and Green, 2007	Foot/ankle – push	60%MVC	420	83
Egana and Green, 2007	Foot/ankle – push	60%MVC	480	86
Egana and Green, 2007	Foot/ankle – push	60%MVC	540	87
Egana and Green, 2007	Foot/ankle – push	60%MVC	600	83
Egana and Green, 2007	Foot/ankle – push	60%MVC	660	90
Egana and Green, 2007	Foot/ankle – push	60%MVC	720	90
Egana and Green, 2007	Foot/ankle – push	60%MVC	780	90
Egana and Green, 2007	Foot/ankle – push	60%MVC	840	90
Egana and Green, 2007	Foot/ankle – push	60%MVC	900	90
Egana and Green, 2007	Foot/ankle – push	60%MVC	960	90
Egana and Green, 2007	Foot/ankle – push	60%MVC	1020	90
Egana and Green, 2007	Foot/ankle – push	60%MVC	1080	88
Egana and Green, 2007	Foot/ankle – push	60%MVC	1140	88
Egana and Green, 2007	Foot/ankle – push	60%MVC	1200	87
Egana and Green, 2007	Foot/ankle – push	80%MVC	1	100
Egana and Green, 2007	Foot/ankle – push	80%MVC	30	96
Egana and Green, 2007	Foot/ankle – push	80%MVC	60	94
Egana and Green, 2007	Foot/ankle – push	80%MVC	90	91
Egana and Green, 2007	Foot/ankle – push	80%MVC	120	90
Egana and Green, 2007	Foot/ankle – push	80%MVC	150	90
Egana and Green, 2007	Foot/ankle – push	80%MVC	180	88
Egana and Green, 2007	Foot/ankle – push	80%MVC	210	90
Egana and Green, 2007	Foot/ankle – push	80%MVC	240	86
Egana and Green, 2007	Foot/ankle – push	80%MVC	270	85
Egana and Green, 2007	Foot/ankle – push	80%MVC	300	86
Egana and Green, 2007	Foot/ankle – push	80%MVC	330	84
Egana and Green, 2007	Foot/ankle – push	80%MVC	360	86
Egana and Green, 2007	Foot/ankle – push	80%MVC	390	82
Egana and Green, 2007	Foot/ankle – push	80%MVC	420	82
Egana and Green, 2007	Foot/ankle – push	80%MVC	450	85
Egana and Green, 2007	Foot/ankle – push	80%MVC	480	84
Egana and Green, 2007	Foot/ankle – push	80%MVC	510	86
Egana and Green, 2007	Foot/ankle – push	80%MVC	540	87
Egana and Green, 2007	Foot/ankle – push	80%MVC	570	80
Egana and Green, 2007	Foot/ankle – push	80%MVC	600	79
Egana and Green, 2007	Foot/ankle – push	80%MVC	630	79
Egana and Green, 2007	Foot/ankle – push	90%MVC	1	100
Egana and Green, 2007	Foot/ankle – push	90%MVC	30	95

Egana and Green, 2007	Foot/ankle – pusł	90%MVC	60	92
Egana and Green, 2007	Foot/ankle – pusł	90%MVC	90	90
Egana and Green, 2007	Foot/ankle – pusł	90%MVC	120	90
Egana and Green, 2007	Foot/ankle – pusł	90%MVC	150	89

		time (sek)	Force (%MVC)
Fulco et al., 1994	Adductor pollicis	1	100
Fulco et al., 1994	Adductor pollicis	60	84
Fulco et al., 1994	Adductor pollicis	120	76
Fulco et al., 1994	Adductor pollicis	180	70

		time (s)	Force (N)
Gonzales and Scheuermann, 2007	Females Handgrip	1	341,5
Gonzales and Scheuermann, 2007	Females Handgrip	120	280
Gonzales and Scheuermann, 2007	Females Handgrip	793,3	180
Gonzales and Scheuermann, 2007	Males Handgrip	1	488,5
Gonzales and Scheuermann, 2007	Males Handgrip	120	390
Gonzales and Scheuermann, 2007	Males Handgrip	684,8	240

		number of contracti	Force (%MVC)
Hamada et al., 2003	G1	1	100
Hamada et al., 2003	G1	2	97
Hamada et al., 2003	G1	3	96
Hamada et al., 2003	G1	4	87
Hamada et al., 2003	G1	5	90
Hamada et al., 2003	G1	6	90
Hamada et al., 2003	G1	7	82
Hamada et al., 2003	G1	8	83
Hamada et al., 2003	G1	9	83
Hamada et al., 2003	G1	10	81
Hamada et al., 2003	G1	11	82
Hamada et al., 2003	G1	12	81
Hamada et al., 2003	G1	13	78
Hamada et al., 2003	G1	14	78
Hamada et al., 2003	G1	15	74
Hamada et al., 2003	G1	16	77
Hamada et al., 2003	G1	16	77
Hamada et al., 2003	GII	1	100
Hamada et al., 2003	GII	2	97
Hamada et al., 2003	GII	3	95
Hamada et al., 2003	GII	4	81
Hamada et al., 2003	GII	5	78
Hamada et al., 2003	GII	6	73
Hamada et al., 2003	GII	7	71

Hamada et al., 2003	GII	8	70
Hamada et al., 2003	GII	9	68
Hamada et al., 2003	GII	10	65
Hamada et al., 2003	GII	11	61
Hamada et al., 2003	GII	12	59
Hamada et al., 2003	GII	13	54
Hamada et al., 2003	GII	14	50
Hamada et al., 2003	GII	15	58
Hamada et al., 2003	GII	16	54
Hamada et al., 2003	GII	16	50

		number of contracti	Torque [Nm]
Mitsukawa et al., 2009	Plantar flexion	1	180
Mitsukawa et al., 2009	Plantar flexion	5	175
Mitsukawa et al., 2009	Plantar flexion	10	170
Mitsukawa et al., 2009	Plantar flexion	15	160
Mitsukawa et al., 2009	Plantar flexion	20	150
Mitsukawa et al., 2009	Plantar flexion	25	140
Mitsukawa et al., 2009	Plantar flexion	30	125
Mitsukawa et al., 2009	Plantar flexion	35	120
Mitsukawa et al., 2009	Plantar flexion	40	115
Mitsukawa et al., 2009	Plantar flexion	45	110
Mitsukawa et al., 2009	Plantar flexion	50	100
Mitsukawa et al., 2009	Plantar flexion	55	100
Mitsukawa et al., 2009	Plantar flexion	60	95

		number of contracti	Force (%MVC)
Morse et al., 2008	Knee extension	1	100
Morse et al., 2008	Knee extension	2	100
Morse et al., 2008	Knee extension	3	100
Morse et al., 2008	Knee extension	4	100
Morse et al., 2008	Knee extension	5	103
Morse et al., 2008	Knee extension	6	99
Morse et al., 2008	Knee extension	7	98
Morse et al., 2008	Knee extension	8	95
Morse et al., 2008	Knee extension	9	94
Morse et al., 2008	Knee extension	10	95
Morse et al., 2008	Knee extension	11	95
Morse et al., 2008	Knee extension	12	94
Morse et al., 2008	Knee extension	13	96
Morse et al., 2008	Knee extension	14	94
Morse et al., 2008	Knee extension	15	93
Morse et al., 2008	Knee extension	16	94
Morse et al., 2008	Knee extension	17	96
Morse et al., 2008	Knee extension	18	96
Morse et al., 2008	Knee extension	19	95

Morse et al., 2008	Knee extension	20	92
Morse et al., 2008	Knee extension	21	89
Morse et al., 2008	Knee extension	22	93
Morse et al., 2008	Knee extension	23	93
Morse et al., 2008	Knee extension	24	92
Morse et al., 2008	Knee extension	25	95
Morse et al., 2008	Knee extension	26	94
Morse et al., 2008	Knee extension	27	93
Morse et al., 2008	Knee extension	28	91
Morse et al., 2008	Knee extension	29	91
Morse et al., 2008	Knee extension	30	92
Morse et al., 2008	Knee extension	31	90
Morse et al., 2008	Knee extension	32	92
Morse et al., 2008	Knee extension	33	91
Morse et al., 2008	Knee extension	34	90
Morse et al., 2008	Knee extension	35	89
Morse et al., 2008	Knee extension	36	89
Morse et al., 2008	Knee extension	37	85
Morse et al., 2008	Knee extension	38	89
Morse et al., 2008	Knee extension	39	89
Morse et al., 2008	Knee extension	40	88
Morse et al., 2008	Knee extension	41	87
Morse et al., 2008	Knee extension	42	88
Morse et al., 2008	Knee extension	43	88
Morse et al., 2008	Knee extension	44	88
Morse et al., 2008	Knee extension	45	88
Morse et al., 2008	Knee extension	46	88
Morse et al., 2008	Knee extension	47	87
Morse et al., 2008	Knee extension	48	88
Morse et al., 2008	Knee extension	49	86
Morse et al., 2008	Knee extension	50	86
Morse et al., 2008	Knee extension	51	86
Morse et al., 2008	Knee extension	52	84
Morse et al., 2008	Knee extension	53	84
Morse et al., 2008	Knee extension	54	85
Morse et al., 2008	Knee extension	55	84
Morse et al., 2008	Knee extension	56	81
Morse et al., 2008	Knee extension	57	83
Morse et al., 2008	Knee extension	58	82
Morse et al., 2008	Knee extension	59	81
Morse et al., 2008	Knee extension	60	83

		number of contracti	Torque (Nm)
Muthalib et al.., 2010	Elbow flexion	1	45
Muthalib et al.., 2010	Elbow flexion	2	43
Muthalib et al.., 2010	Elbow flexion	3	41
Muthalib et al.., 2010	Elbow flexion	4	41
Muthalib et al.., 2010	Elbow flexion	5	40

Muthalib et al.., 2010	Elbow flexion	6	39
Muthalib et al.., 2010	Elbow flexion	7	38
Muthalib et al.., 2010	Elbow flexion	8	38
Muthalib et al.., 2010	Elbow flexion	9	38
Muthalib et al.., 2010	Elbow flexion	10	37
Muthalib et al.., 2010	Elbow flexion	11	37
Muthalib et al.., 2010	Elbow flexion	12	36
Muthalib et al.., 2010	Elbow flexion	13	37
Muthalib et al.., 2010	Elbow flexion	14	36
Muthalib et al.., 2010	Elbow flexion	15	35
Muthalib et al.., 2010	Elbow flexion	16	35
Muthalib et al.., 2010	Elbow flexion	17	35
Muthalib et al.., 2010	Elbow flexion	18	35
Muthalib et al.., 2010	Elbow flexion	19	34
Muthalib et al.., 2010	Elbow flexion	20	33
Muthalib et al.., 2010	Elbow flexion	21	34
Muthalib et al.., 2010	Elbow flexion	22	33
Muthalib et al.., 2010	Elbow flexion	23	32
Muthalib et al.., 2010	Elbow flexion	24	31
Muthalib et al.., 2010	Elbow flexion	25	31
Muthalib et al.., 2010	Elbow flexion	26	31
Muthalib et al.., 2010	Elbow flexion	27	30
Muthalib et al.., 2010	Elbow flexion	28	31
Muthalib et al.., 2010	Elbow flexion	29	31
Muthalib et al.., 2010	Elbow flexion	30	31
Muthalib et al.., 2010	Elbow flexion	31	31
Muthalib et al.., 2010	Elbow flexion	32	31
Muthalib et al.., 2010	Elbow flexion	33	31
Muthalib et al.., 2010	Elbow flexion	34	31
Muthalib et al.., 2010	Elbow flexion	35	30
Muthalib et al.., 2010	Elbow flexion	36	30
Muthalib et al.., 2010	Elbow flexion	37	30
Muthalib et al.., 2010	Elbow flexion	38	30
Muthalib et al.., 2010	Elbow flexion	39	30
Muthalib et al.., 2010	Elbow flexion	40	30
Muthalib et al.., 2010	Elbow flexion	41	30
Muthalib et al.., 2010	Elbow flexion	42	30
Muthalib et al.., 2010	Elbow flexion	43	30
Muthalib et al.., 2010	Elbow flexion	44	30
Muthalib et al.., 2010	Elbow flexion	45	30
Muthalib et al.., 2010	Elbow flexion	46	30
Muthalib et al.., 2010	Elbow flexion	47	30
Muthalib et al.., 2010	Elbow flexion	48	30
Muthalib et al.., 2010	Elbow flexion	49	29
Muthalib et al.., 2010	Elbow flexion	50	29

		time (s)	Force (%MVC)
Ordway et al., 1977	Elbow flexion	1	100

Ordway et al., 1977	Elbow flexion	15	91
Ordway et al., 1977	Elbow flexion	30	87
Ordway et al., 1977	Elbow flexion	45	80
Ordway et al., 1977	Elbow flexion	60	77
Ordway et al., 1977	Elbow flexion	75	73
Ordway et al., 1977	Elbow flexion	90	69
Ordway et al., 1977	Elbow flexion	105	66
Ordway et al., 1977	Elbow flexion	120	63
Ordway et al., 1977	Elbow flexion	135	62
Ordway et al., 1977	Elbow flexion	150	60
Ordway et al., 1977	Elbow flexion	165	58
Ordway et al., 1977	Elbow flexion	180	56
Ordway et al., 1977	Elbow flexion	195	55
Ordway et al., 1977	Elbow flexion	210	54
Ordway et al., 1977	Elbow flexion	225	53
Ordway et al., 1977	Elbow flexion	240	52
Ordway et al., 1977	Elbow flexion	255	51
Ordway et al., 1977	Elbow flexion	270	50
Ordway et al., 1977	Elbow flexion	285	50
Ordway et al., 1977	Elbow flexion	300	49
Ordway et al., 1977	Knee extension	1	100
Ordway et al., 1977	Knee extension	15	91
Ordway et al., 1977	Knee extension	30	87
Ordway et al., 1977	Knee extension	45	82
Ordway et al., 1977	Knee extension	60	78
Ordway et al., 1977	Knee extension	75	73
Ordway et al., 1977	Knee extension	90	68
Ordway et al., 1977	Knee extension	105	63
Ordway et al., 1977	Knee extension	120	58
Ordway et al., 1977	Knee extension	135	55
Ordway et al., 1977	Knee extension	150	53
Ordway et al., 1977	Knee extension	165	50
Ordway et al., 1977	Knee extension	180	49
Ordway et al., 1977	Knee extension	195	47
Ordway et al., 1977	Knee extension	210	46
Ordway et al., 1977	Knee extension	225	45
Ordway et al., 1977	Knee extension	240	44
Ordway et al., 1977	Knee extension	255	44
Ordway et al., 1977	Knee extension	270	43
Ordway et al., 1977	Knee extension	285	41
Ordway et al., 1977	Knee extension	300	40

		time (s)	Force (%MVC)
Saito et al., 2008	Males handgrip	1	100
Saito et al., 2008	Males handgrip	30	82
Saito et al., 2008	Males handgrip	60	71
Saito et al., 2008	Males handgrip	90	68
Saito et al., 2008	Males handgrip	120	62

Saito et al., 2008	Males handgrip	180	54
Saito et al., 2008	Males handgrip	240	51
Saito et al., 2008	Females handgrip	1	100
Saito et al., 2008	Females handgrip	30	89
Saito et al., 2008	Females handgrip	60	80
Saito et al., 2008	Females handgrip	90	73
Saito et al., 2008	Females handgrip	120	70
Saito et al., 2008	Females handgrip	180	64
Saito et al., 2008	Females handgrip	240	59

	time (%)	Force (%MVC)
Saugen et al., 1997	1	100
Saugen et al., 1997	25	75
Saugen et al., 1997	50	67
Saugen et al., 1997	75	62
Saugen et al., 1997	100	58

		time (%)	Force (%MVC)
Thomasa and Valle, 2001	Elbow extension	1	100
Thomasa and Valle, 2001	Elbow extension	20	80
Thomasa and Valle, 2001	Elbow extension	40	76
Thomasa and Valle, 2001	Elbow extension	60	68
Thomasa and Valle, 2001	Elbow extension	80	65
Thomasa and Valle, 2001	Elbow extension	100	50

			time (s)	Force (%MVC)
Wallström and Nordenskiöld, 200	Handgrip	Males righ	1	100
Wallström and Nordenskiöld, 200	Handgrip	Males righ	90	68
Wallström and Nordenskiöld, 200	Handgrip	Males righ	180	59
Wallström and Nordenskiöld, 200	Handgrip	Females ri	1	100
Wallström and Nordenskiöld, 200	Handgrip	Females ri	90	66
Wallström and Nordenskiöld, 200	Handgrip	Females ri	180	56
Wallström and Nordenskiöld, 200	Handgrip	Males left	1	100
Wallström and Nordenskiöld, 200	Handgrip	Males left	90	71
Wallström and Nordenskiöld, 200	Handgrip	Males left	180	61
Wallström and Nordenskiöld, 200	Handgrip	Females le	1	100
Wallström and Nordenskiöld, 200	Handgrip	Females le	90	66
Wallström and Nordenskiöld, 200	Handgrip	Females le	180	60

		**Number of contract**	**Force (kg)**
White et al., 2013	Males handgrip	1	45
White et al., 2013	Males handgrip	2	42

White et al., 2013	Males handgrip	3	40
White et al., 2013	Males handgrip	4	40
White et al., 2013	Males handgrip	5	38
White et al., 2013	Males handgrip	6	35
White et al., 2013	Males handgrip	7	35
White et al., 2013	Males handgrip	8	34
White et al., 2013	Males handgrip	9	33
White et al., 2013	Males handgrip	10	30
White et al., 2013	Males handgrip	11	31
White et al., 2013	Males handgrip	12	34
White et al., 2013	Females handgrip	1	27
White et al., 2013	Females handgrip	2	25
White et al., 2013	Females handgrip	3	24
White et al., 2013	Females handgrip	4	23
White et al., 2013	Females handgrip	5	22
White et al., 2013	Females handgrip	6	21
White et al., 2013	Females handgrip	7	20
White et al., 2013	Females handgrip	8	19
White et al., 2013	Females handgrip	9	18
White et al., 2013	Females handgrip	10	17
White et al., 2013	Females handgrip	11	18
White et al., 2013	Females handgrip	12	18
White et al., 2013	Males knee extension	1	268
White et al., 2013	Males knee extension	2	272
White et al., 2013	Males knee extension	3	268
White et al., 2013	Males knee extension	4	268
White et al., 2013	Males knee extension	5	243
White et al., 2013	Males knee extension	6	240
White et al., 2013	Males knee extension	7	238
White et al., 2013	Males knee extension	8	228
White et al., 2013	Males knee extension	9	222
White et al., 2013	Males knee extension	10	215
White et al., 2013	Males knee extension	11	208
White et al., 2013	Males knee extension	12	205
White et al., 2013	Females knee extension	1	155
White et al., 2013	Females knee extension	2	153
White et al., 2013	Females knee extension	3	148
White et al., 2013	Females knee extension	4	146
White et al., 2013	Females knee extension	5	145
White et al., 2013	Females knee extension	6	141
White et al., 2013	Females knee extension	7	140
White et al., 2013	Females knee extension	8	140
White et al., 2013	Females knee extension	9	140
White et al., 2013	Females knee extension	10	138
White et al., 2013	Females knee extension	11	135
White et al., 2013	Females knee extension	12	132
